# Two Years Follow up of Domain Specific Cognitive Training in Relapsing Remitting Multiple Sclerosis: A Randomized Clinical Trial

**DOI:** 10.3389/fnbeh.2016.00028

**Published:** 2016-02-23

**Authors:** Flavia Mattioli, Fabio Bellomi, Chiara Stampatori, Leandro Provinciali, Laura Compagnucci, Antonio Uccelli, Matteo Pardini, Giuseppe Santuccio, Giuditta Fregonese, Marianna Pattini, Beatrice Allegri, Raffaella Clerici, Annalisa Lattuada, Cristina Montomoli, Barbara Corso, Paolo Gallo, Alice Riccardi, Angelo Ghezzi, Marco Roscio, Maria Rosaria Tola, Chiara Calanca, Daria Baldini, Debora Trafficante, Ruggero Capra

**Affiliations:** ^1^Neuropsychology Unit, Spedali Civili of BresciaBrescia, Italy; ^2^Clinica Neurologica, University of AnconaAncona, Italy; ^3^Clinica Neurologica, MS Center, University of GenovaGenova, Italy; ^4^Neurology Unit, Azienda Ospedaliera Valtellina ValchiavennaSondrio, Italy; ^5^Neurology Unit, Fidenza HospitalFidenza, Italy; ^6^Neurology Unit, Como HospitalComo, Italy; ^7^Biostatistics Unit, Department of Public Health, Experimental and Forensic Medicine, Pavia UniversityPavia, Italy; ^8^National Research Council, Neuroscience InstitutePadova, Italy; ^9^Clinica Neurologica, University of PadovaPadova, Italy; ^10^UO Neurologia, MS Center, Gallarate HospitalGallarate, Italy; ^11^UO Neurologia, Ferrara UniversityFerrara, Italy; ^12^UO Neurologia, Lecco HospitalLecco, Italy; ^13^Multiple Sclerosis Center, Spedali Civili of BresciaBrescia, Italy

**Keywords:** multiple sclerosis, cognitive rehabilitation, randomize clinical trial, neuropsychology

## Abstract

Cognitive rehabilitation in multiple sclerosis (MS) has been reported to induce neuropsychological improvements, but the persistence of these effects has been scarcely investigated over long follow ups. Here, the results of a multicenter randomized clinical trial are reported, in which the efficacy of 15 week domain specific cognitive training was evaluated at 2 years follow up in 41 patients. Included patients were randomly assigned either to domain specific cognitive rehabilitation, or to aspecific psychological intervention. Patients who still resulted to be cognitively impaired at 1 year follow up were resubmitted to the same treatment, whereas the recovered ones were not. Neuropsychological tests and functional scales were administered at 2 years follow up to all the patients. Results revealed that both at 1 and at 2 years follow up more patients in the aspecific group (18/19, 94% and 13/17, 76% respectively) than in the specific group (11/22, 50% and 5/15, 33% respectively) resulted to be cognitively impaired. Furthermore patients belonging to the specific group showed significantly less impaired tests compared with the aspecific group ones (*p* = 0.02) and a significant amelioration in the majority of the tests. On the contrary patients in the aspecific group did not change. The specific group subjects also perceived a subjective improvement in their cognitive performance, while the aspecific group patients did not. These results showed that short time domain specific cognitive rehabilitation is a useful treatment for patients with MS, shows very long lasting effects, compared to aspecific psychological interventions. Also subjective cognitive amelioration was found in patients submitted to domain specific treatment after 2 years.

## Introduction

Patients affected with multiple sclerosis (MS) often have a certain degree of cognitive impairment. Approximately 40–70% of patients present a certain degree of cognitive deficit, independently from disease duration, disease severity and physical disability (Chiaravalloti and DeLuca, 2008). This frequently contributes to the loss of employment, reduced social and working abilities and worsened quality of life (Pompeii et al., 2005; Putzki et al., 2009). The main affected cognitive areas are attention, information processing speed, memory and executive functions (Calabrese, 2006; Chiaravalloti and DeLuca, 2008; Duque et al., 2008; Prakash et al., 2008). The spontaneous evolution of cognitive deficits in MS is known to be worsening, as shown by a 10 years longitudinal study conducted on patients who were in large measure untreated, reporting the increase in the number of moderately or severely impaired MS patients and a reduction of mildly impaired ones over time. Among clinical predictors, incipient cognitive decline seems to be the major risk factor for further patents’ deterioration in the short-term. In the long-term, the likelihood increases that also patients with initial cognitive preservation may deteriorate (Amato et al., 2006b).

Although cognitive deficits are prominent and detrimental in MS, surprisingly few studies investigated the effectiveness of cognitive training (Jønsson et al., 1993; Plohmann et al., 1998; Fink et al., 2010; Mattioli et al., 2010a; Amato et al., 2014; Chiaravalloti and DeLuca, 2015). Despite differences emerged between the results of early published studies—mainly depending on methodological issues, such as clinical heterogeneity across patients in terms of disability, type of cognitive impairment, type of MS and type of immunomodulatory drug they were prescribed (Thomas et al., 2006)—the most recent investigations provided convergent support to the usefulness of cognitive rehabilitation in MS (Rosti-Otajärvi and Hämäläinen, 2014). Particularly, episodic memory (Chiaravalloti et al., 2013), autobiographical memory (Ernst et al., 2015) and attention abilities (Mattioli et al., 2010a, 2012; Amato et al., 2014) and executive functions (Mattioli et al., 2010a) have been shown to significantly improve after a domain specific cognitive training in randomized clinical trials—either compared to no treatment or to a control treatment. The positive effects of these cognitive trainings have been reported both immediately after the end of the treatment (Mattioli et al., 2010b; Hubacher et al., 2015) and 6 months after the end (Mattioli et al., 2012; Chiaravalloti et al., 2013; Rosti-Otajärvi et al., 2013). An independent improvement also in depression—a frequently associated disorder in MS—and quality of life have also been shown after cognitive interventions (Mattioli et al., 2010a, 2012).

However, the above mentioned studies were all performed by one single center and only in one recent study (Mattioli et al., 2015) a multicenter approach has been used, providing supporting evidences of the reliability of the domain specific approach (Sprague et al., 2009). This study, the Sclerosi Multipla Intensive Cognitive Training (SMICT) is a multicenter randomized Italian clinical trial on relapsing remitting (RR) patients. It was aimed at comparing the efficacy of a domain specific cognitive training with a non specific psychological treatment over 2 years follow up. Preliminary data of this collaborative study with the results of the 1st year follow up, showed that patients treated with the domain specific approach had a significantly lower number of impaired cognitive tests and resulted to be cognitively recovered in a significantly higher proportion compared to those ones submitted to the non-specific psychological intervention (Mattioli et al., 2015). Furthermore, all the patients of the study were prescribed the same immunomodulatory drug, (in fact, different therapeutic regimens in previous trials could have been a confounding variable). Through the persistence at 1 year of the positive effects of domain specific cognitive interventions has been published, the exact need for treatment beyond the 1st year of follow up still needs to be further investigated and the possible beneficial effect of repeated boosters of cognitive training in MS patients still needs to be investigated as well.

The aim of the current article is to provide final results of the SMICT study over 2 years follow up in MS, evaluating the persistence over 2 years of cognitive improvement induced by the domain specific cognitive training. Also the possible efficacy of a repeated cognitive treatment in the 2nd year of follow up will be examined.

## Materials and Methods

### Subjects

The Randomized Clinical Trial (Spedali Civili of Brescia trial Register NP: 560) was performed according to the Helsinki Declaration and after the approval of the Ethical Committee (Comitato Etico Provinciale di Brescia, January 2010). Patients’ enrolment started on June 2010 and ended 31 December 2011. It involved 10 MS centers in Italy. Patients affected with MS, according to Poser and Brinar (2001) criteria with a RR course were included in the study, after their signed informed consent was obtained. To participate in the study, all patients needed to have been prescribed interferon beta 1A 44 mcg 3 times/week no later than 6 months before, in order to have the same drug regimen in patients. This first line therapeutic regimen was chosen, as it has been shown to be effective on several neuropsychological measures (Amato et al., 2013). Patients were included only if impaired (age corrected *z* score < −1.5 SD to norms) in at least one of the tests included in the Italian version of the Rao Brief Repeatable Battery and Stroop test. Exclusion criteria were dementia (excluded by means of anamnestic reports as well as MMSE >24 in patients), previous or present psychiatric disorders (requiring pharmacological treatment) and clinically evident relapse in the previous 6 months. For the included patients, the disease duration, the disability in the Expanded Disability Status Scale (EDSS; Kurtzke, 1983), the relapse rate and steroid consumption (grams of intra venous methylprednisolone) in the previous year were registered.

### Neuropsychological Evaluations

Three neuropsychological evaluations were performed for each patient: T0 at baseline before enrolment, T12 after 1 year and T24 after 2 years from the baseline. The Italian version of the Rao’s Brief Repeatable Battery (Amato et al., 2006a), including Paced Auditory Serial Addition Task (PASAT 2″, PASAT 3″), Simbol Digit Modality Test (SDMT), Spatial Recall Test (SPART) 10/36 and Delayed Recall (SPART D), Selective Reminding Test Long Term storage (SRT LTS), Consistent Long Term Retrieval (SRT CLTR), Delayed Recall (SRT DR), the Controlled Oral Words Association (COWA) with the Phoneme (P) and Category (C) modalities (Mattioli et al., 2010a) and Stroop test (Barbarotto et al., 1998). Alternative forms, when available, were used, in order to avoid test retest effects and learning effects (Goretti et al., 2014). All the tests were corrected by age and education, according to published norms. A test was considered impaired, if its corrected score fell below −1.5 SD.

In addition, three functional scales were administered, in order to evaluate the fatigue (Modified Fatigue Impact Scale, mFIS; Kos et al., 2006), the possible deflection of mood (Montgomery-Asberg Depression Rating Scale, MADRS; Montgomery and Asberg, 1979) and the quality of life (Multiple Sclerosis Quality of Life Questionnaire, MSQoL; Solari et al., 1999) at the same intervals.

In order to measure the patients’ subjective perception of cognitive amelioration after treatment, the item 6 of MADRS has been selected and used: it requires the patient to rate on a 6 point Likert scale his/her difficulties in collecting one’s thoughts, where 0 means “no difficulty in concentrating” and 6 means “unable to read or converse without great difficulty” and compared between T0 and T24.

### Treatments

Patients were randomly assigned to Specific Treatment Group (SG) or to Aspecific Treatment Group (AG), for 15 consecutive weeks with 2 weekly 60’ sessions. Randomization (according to a computer-generated list of random number) and statistical analysis of data were carried out by an independent center, from whom all the Centers received the patients’ number.

### Specific Treatment

Specific treatment was administered according to the impaired neuropsychological function: Plan a Day software of the Rehacom^1^ was used if a patient resulted impaired in executive functions (that is if his/her poor score was in the Stroop test or in the COWA P or COWA/C); Memory software of the same package was used if the patient was impaired in either the SRT or SPART verbal or spatial memory measures and the previously described 29 A/IP training, if he/she resulted impaired in attention/speeded information processing domain (pathological PASAT 2″, PASAT 3″, SDMT). If a patient was impaired in more than one domain, all the single domain trainings were balanced in the hourly session each time. Exercises complexity was adapted each time to the severity of each single patient’s impairment in the selected domain, with the aim that the exercise had to be challenging in each treatment session.

#### Plan a Day

The Plan a Day procedure trains the patient’s ability to organize, plan and develop solution strategies, employing realistic simulations of a set of scheduled dates and duties to be organized at specific places in a virtual small city map. Times for planning and schedules are registered for each patient at each session and only improvement and acquisition of sufficient planning abilities for fulfilling all the appointments required led to an improved level in the following treatment session. Fifty four levels of increasing complexity are available, in order to challenge any grade of impairment. This was considered a strategic behavior acquisition. For further description of the treatment see Mattioli et al. (2015).

#### Memory

Patients were asked to give answer to multiple choice or open questions about tales of increasing length, which were presented on the PC, whose complexity was chosen on the basis of the patient’s memory impairment. Ten levels of difficulty—also with interfering condition of two or three tales alternatively presented with the other tales’ questions—were progressively presented to the patients.

#### A/IP Training

A specific speeded information training with increasing velocity (from 4000 to 1800 ms interval), which has been shown to be effective in patients with brain injuries, was used, consisting of a modified PASAT task with numbers, words and months of the year, according to Serino et al. (2006) procedure.

### Aspecific Training

The A treatment (not domain specific intervention, but a generic psychological intervention, considered as control treatment) was conducted by the psychologist addressing the following items with the patient: the patient’s disease perception (with the aid of scientific articles dealing with MS), eventual limitation in the patient’s occupation due to MS, possible difficulties on his/her job, problems with the patient’s family life and leisure activities, specific problems of the patient’s due to MS (i.e., sexual, affective). The aim of this sort of psycho education was not to specifically treat a cognitive ability, but rather to discuss with the patient about the functional impairment due to MS, avoiding to treat depression or to have any behavioral or psychoanalytic approach. This type of psycho educational treatment, considered as a control treatment have been accepted as ethical by the Ethical Committees of all the Centers, as no sure evidences exist till now about the superiority of domain specific treatments of memory, attention and executive functions on the aspecific psychological approaches in MS.

All the treating psychologists were trained by attending 10 consecutive training meetings with the psychologists of the coordinator center.

The same treatment administered after randomization was repeated in the 2nd year of the study, after T12 evaluation, only if a patient resulted to be still impaired in at least one neurpsychological test.

Patients’ neuropsychological evaluations and treatments were done by different neuropsychologists and performed in a quiet room, according with standardized published procedures, with maximum attention paid by the neuropsychologists in order to avoid interference from possible low motivation of patients on performance. All the patients were reminded about the study protocol in each session, in order to refresh the context of the evaluations.

### Statistical Analysis

A sample size of 14 patients for each group was necessary (Faul et al., 2007) in order to have a 5% significance level and a 90% statistical power. Descriptive statistics are expressed as median and/or means ± SD. Due to the nature of the variables and the sample size, non-parametric tests were performed. The two patients’groups were compared using Mann-Whitney’s statistic test for quantitative variables and Fisher’s exact test for qualitative variables.

Pearson chi-squared test was applied to qualitative data. Repeated measures within group were evaluated by Wilcoxon signed-rank test (over two-time point) and by Friedman’s test (over the three-time point). Repeated measures mixed models were applied to each variable to take into consideration simultaneously the effect of treatment, time and their interaction. All statistical analyses were performed using STATA/SE version 12.1 software and a *p* < 0.05 was considered significant. A Poisson multivariate regression model has been used to analyze the relationship between number of pathological tests and treatment, using EDSS as a covariate.

## Results

**Figure 1:**
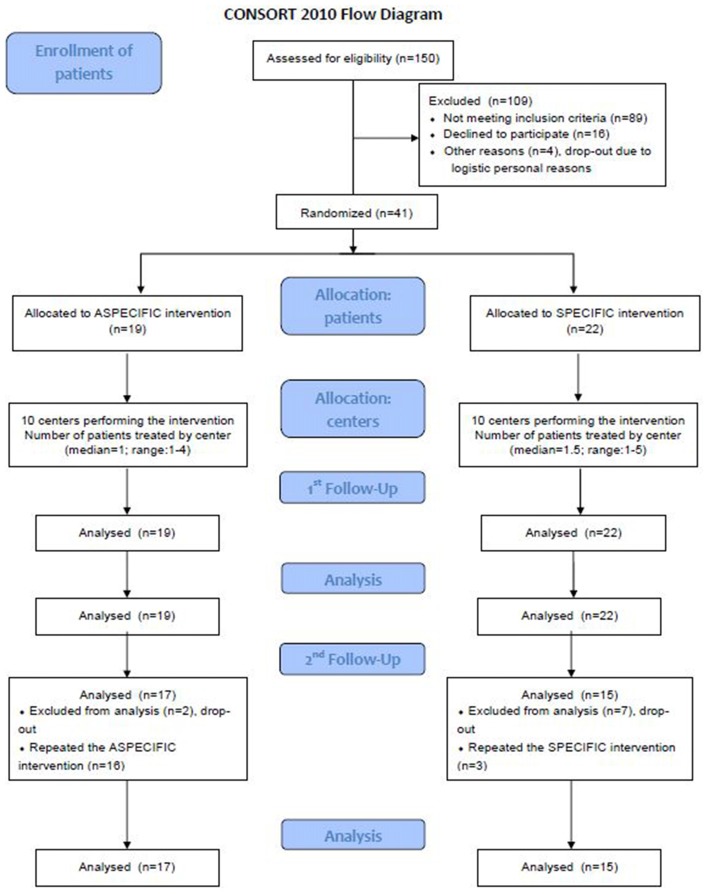
**Consort diagram of the study**.

The AG consisted of 19 patients both at T0 and at T12. Eighteen of them resulted to be still impaired at T12 (that is they had at least one impaired test in the neuropsychological battery), two of which refused to repeat the neuropsychological evaluation at T24, so 16 patients in the AG repeated the aspecific treatment. In the SG, which at T0 and T12 consisted of 22 subjects, of whom 11 were still impaired at T12, 8 subjects refused to repeat the neuropsychological evaluation and only three patients repeated the treatment (with the same domain specific intervention as in the 1st year). Figure 1 reports the CONSORT diagram. Comparing the number of patients who were still cognitively impaired at T12 between groups, a significantly higher number in the AG than in SG was found (16/17, 94% vs. 3/15, 20%, Pearson test *p* = 0.014). Also at T24 a significantly higher number (13/17) of patients in the AG than in the SG (5/15) resulted to be still cognitively impaired (Pearson test *p* = 0.014). This sample was considered for statistical analysis of single tests’ scores.

As previously reported (Mattioli et al., 2015) baseline (T0) clinical characteristics of patients did not differ between groups in terms of disease duration, age, education, EDSS, steroid consumption and number of relapses. At T12 only EDSS score was changed between groups, which resulted to be higher in patients submitted to the Aspecific Treatment compared with patients submitted to the Specific Treatment (Table 1). Within group change revealed a worsened EDSS in AG at T24 (mean EDSS 3.47, SD 1.76; repeated measures mixed models: significant effect of both group and time, as well as interactions: *p* = 0.003, *p* = 0.019, *p* = 0.002) and an unchanged EDSS in SG. However, the number of relapses was not significantly different between the two groups at T24 (Mann Whitney *p* = 0.99), with a decrease compared with the number in the previous year in both groups. Similarly, steroid consumption decreased in the 2nd year follow up, not significantly different between groups (Mann Whitney test *p* = 0.3), indicating an overall similarity between disease physical severity between the groups.

**Table 1:** **Characteristics of patients at T12**.

	**AG (*n* = 17)**		**SG (*n* = 15)**		*p*-value*
	Median	Mean ± SD	Median	Mean ± SD
Age (years)	49	44.88 ± 9.96	47	44.80 ± 8.69	0.75
Years of education	13	12.12 ± 3.62	10	10.93 ± 3.17	0.41
Disease duration (months)	60	87.18 ± 74.83	30	67.20 ± 88.77	0.10
Number of relapses (previous year)	0	0.53 ± 0.94	0	0.33 ± 0.62	0.72
EDSS	3	2.97 ± 1.49	2	1.63 ± 0.95	0.0094
Steroid (gr)^#^	0	2.29 ± 4.40	0	0.71 ± 1.82	0.30

*Mann-Whitney test. AG, Aspecific Treatment Group; SG, Specific Treatment Group; EDSS, Expanded Disability Status Scale. ^#^Methylprednisolone consumption in the 1st year of the study.

As shown in Table 2 the number of impaired tests in AG was not significantly changed between T0 and T24 (*p* = 0.51), while in the SG a significant reduction of impaired tests was observed (*p* < 0.001). Considering the comparison between AG and SG also at T12 in the number of impaired tests a significant reduction of them was found in the SG (Table 2). In addition, a between group comparisons of the number of pathological tests at T24 further confirms less impaired tests in SG compared with the AG (0.75 ± 1.34, 2.29 ± 2.52, *p* = 0.02). However, in a multivariate analysis, taking into account EDSS as a covariate, the difference in the number of pathological tests between AG and SG group loses its statistical significance (*p* = 0.089).

**Table 2:** Number of pathological tests at baseline (T0) and after rehabilitation (T24), and between groups comparison (*) at T24.

	AG (*n* = 17)				SG (*n* = 15)				
	T0	T12^∧^	T24		T0	T12	T24
	*m*	*m*	*m*	*p*-value**	*m*	*m*	*m*	*p*-value**	*p*-value*
Number of pathological tests	2	3	2	0.5169	2	0	0	*0.0006*	*0.0162*

**Wilcoxon signed-rank test. *Mann-Whitney test at T24. ^∧^Wilcoxon signed rank test: AG T0 vs. T24 p = 0.51; SG T0 vs. T24 p = 0.006.

On the other hand, no differences emerged in MSQoL, mFIS and MADRS between groups at T24 (Table 3).

**Table 3:** **T24 MSQoL, mFIS and MADRS scores in AG and SG**.

	AG (*n* = 17) median	SG (*n* = 15) median	*p*-value*
MSQoL	140	161	0.14
mFIS	32	20	0.27
MADRS	5	5	0.34
EDSS	3	1.5	0.005

*Mann-Whitney test at T24.

Also single tests’ performance at T24 revealed significantly better performances of SG group compared to AG in SDMT (35 vs. 46, *p* = 0.02) and COWAL (30 vs. 35, *p* = 0.006), with a trend toward significance in SPART DR (five vs. seven, *p* = 0.055). Subjects in SG significantly improved their scores in almost all neuropsychological tests after both 1 and 2 years (Table 4), whereas subjects in AG did not significantly improved their scores in none of the tests. In repeated measures mixed models, interactions between treatment and time was statistically significant for PASAT 3″, SPART DR, SDMT and Stroop test and marginally significant for SPART 10/36 (*p* = 0.0649) and SRT LTS (*p* = 0.0556). These results confirm the significant difference on the neuropsychological tests between the two groups at the different follow ups, showing differences at T12 and at T24. Noteworthy, considering only the three patients of the SG, who, as being impaired at T12, were submitted to repeated rehabilitation the second time, they resulted to be unchanged at T24, in terms of number of impaired tests. The patients were all impaired in memory (of them one also impaired in attention and another in executive function), but, due to the small number, no statistical analysis on the effects of the training between domains was carried on.

**Table 4:** **Comparison of neuropsychological tests median raw scores at baseline (T0), after 1 year rehabilitation (T12) and after 2-years rehabilitation (T24)**.

	**AG (*n* = 17)**			**SG (*n* = 15)**			T 24: AG vs. SG
	T0	T12	T24	T0	T12	T24	*p*-value*
PASAT3	37	36	38	36	45	44	0.1619
PASAT2	23	29	30	24	35	32	0.1302
SPART10/36	18	19	19	15	22	21	0.1497
SPARTDR	6	6	5	4	7	7	0.0559
SRTLTS	33	40	37	30	44	46	0.2487
SRTCLTR	24	28	26	23	34	33	0.5966
SRTDR	8	8	8	7	9	8	0.7741
SDMT	40	40	35	44	49	46	0.0256
COWAL	28	30	30	34	35	35	0.0068
COWAC	40	42	41	38	42	45	0.3155
Stroop	20	27	25	23	30	30	0.1441

*Mann Whitney test. Between groups comparison (*) at T24.

Although the total MADRS score did not differ between groups, the results on MADRS item 6 measuring the subjective perception of cognitive deficits, resulted to be significantly reduced in SG (median T0 2, median T24 0; *p* = 0.0182 Wilcoxon signed rank test) and unchanged in the AG (median T0 2, median T24 2; *p* = 0.88 Wilcoxon signed rank test) and to be significantly better in SG than in the AG at T24 (*p* = 0.291 Mann Whitney test), showing a better subjective perception of cognitive performance in SG.

## Discussion

The main result of the present study is that domain specific cognitive rehabilitation can be effective and can sustain significant cognitive improvements up to 2 years in patients with RR MS. Specific exercises aimed at treating the impaired cognitive domain are shown to induce significantly better results both on cognition and on subjective perception of cognitive impairment in patients, compared with non domain specific psychological interventions. Results showed the greater amelioration both considering the reduction in the number of impaired neuropsychological tests and the improvement in single tests’ scores over time. Particularly, nearly all (94%) patients assigned to aspecific treatment and only 20% of those assigned to the specific treatment, needed the repeated rehabilitation in the 2nd year of follow up. This indicates that the domain specific intervention provided in the 1st year caused beneficial effects lasting up to 2 years. Notably, the only three patients, who—being still impaired at T12—needed a repeated treatment, did not change in severity their cognitive impairment, measured as the number of impaired tests. The uselessness of repeated treatments in neuropsychological rehabilitation of MS is in line—although with longer time of follow up—with the conclusions of Chiaravalloti et al. (2013), who found no effects of repeated booster sessions of memory rehabilitation in their study.

Moreover, after 2 years, patients assigned to the SG showed fewer impaired neuropsychological tests compared to those assigned to AG and also had a significantly better performance in tests measuring information processing speed and executive functions. Finally SG patients perceived a subjective improvement in their cognitive performance, whereas AG patients did not. This finding is relevant in MS, as shows that appropriately conducted cognitive rehabilitation can be able to reduce the worsening spontaneous evolution of cognitive impairment of MS patients and beneficially impact on their disease relate disability over time.

The cognitive improvement found in SG is, in our opinion, only ascribed to the type of the treatment assigned, as the other clinical variables were not different between groups, as well as the type of the pharmacological treatment used and the disease severity. Although, it is worth noting that a possibly higher disease activity in AG compared to SG cannot be totally ruled out: throughout the study, EDSS—relatively low in both groups—worsened in AG and remained substantially stable in SG. On the other hand steroid consumption and relapse rates were not different between groups across 2 years follo up. Furthermore, it is known that EDSS relies more on physical than on cognitive disability; so it is possible that, at individual level, physical disability worsened more in AG patients due to less response to immunomodulatory drug instead of more active disease. Furthermore, a different response to interferon can be hypothesized in AG compared to SG and also possible spinal or cerebellar new lesions (that would not be relevant under a cognitive point of view) could have also been responsible for motor/EDSS worsening). Overall, it is reasonable to conclude that—although neuroradiological data on new lesions are missing in this study-, disease activity relevant for cognitive worsening can reasonably be considered similar between groups; not the same for motor disability.

The possibility of successfully rehabilitate MS patients’ cognitive impairment with domain specific PC assisted, replicable and easily to administer rehabilitative programs in the clinical setting, with long standing results up to 2 years, has never been demonstrated till now. This prompts future research with larger samples of patients, as the main limitation of our study is the low number of included MS subjects. The main reasons of this, is in our opinion, the inclusion criteria and probably for some MS centers, the logistic problems met by patients whose psychologist was accessible within the Hospital, compared with those who met the psychologist outside, in rehabilitation clinics. Centers whose neuropsychologist was easily accessible in rehabilitative structures had in fact greater inclusions and less drop outs. A future issue will be the possibility of structuring home based trainings, monitoring the effective practice by each patients, both in terms of correctness and of number of exercises performed at home. This could give additional interesting data on the effects of intensive cognitive rehabilitation in MS.

Another limitation of this study is the type of the control aspecific treatment. Although in randomized clinical trials dealing with neuropsychological rehabilitation, a psychological control treatment is difficult to be set and deserves intrinsic limitation, it is overtly recognized to be useful and recommended as a comparator treatment (Rosti-Otajärvi and Hämäläinen, 2014). The intrinsic limitation of a non domain specific psychological control treatment relies mainly in the fact that patients submitted to it may well have become aware of the psycho educational nature of this treatment, and may have consciously or unconsciously inferred that they were receiving the placebo treatment. This, in the specific case of our study, may have mainly impact the subjective perception of cognitive improvement we found in AG, more than the objective neuropsychological evaluation at follow ups.

In this study, similarly to others (Hämäläinen and Rosti-Otajärvi, 2014; Chiaravalloti and DeLuca, 2015) not only objective neuropsychological tests, but also subjective perception of the patients’ cognitive improvement was measured. Patients in the SG subjectively perceived higher improvement in cognitive abilities than AG, although the scale we used was relatively simple and in the future better functional scales are welcome.

In conclusion, despite some limits, this is the first study evaluating the persistence of the cognitive improvement induced by a domain specific cognitive rehabilitation in MS with a follow up of 2 years. Despite limitations, results interestingly show a significant effect of this treatment in a multi center setting and its persistence after 2 years.

## Author Contributions

FM: Principal investigator, study design, results interpretation, manuscript writing and revision. CM and BC: Statistical analysis of the data. CS: Data collection from all the Centers and patients’ evaluation in Brescia Center. FB: Treating psychologist in Brescia. RC: Patients selection in Brescia. AU, MP: Patients selection, patients evaluation and treatment in Genova. LP, LC: Patients selection, patients evaluation and treatment in Ancona. PG, AR: Patients selection, patients evaluation and treatment in Padova. GS, GF: Patients selection, patients evaluation and treatment in Sondrio. MP, BA: Patients selection, patients evaluation and treatment in Fidenza. RC, AL: Patients selection, patients evaluation and treatment in Como. MRT, CC: Patients selection, patients evaluation and treatment in Ferrara. AG, MR: Patients selection, patients evaluation and treatment in Gallarate. We thank Fondazione Cesare Serono for research suport.

## Conflict of Interest Statement

The authors declare that the research was conducted in the absence of any commercial or financial relationships that could be construed as a potential conflict of interest. The reviewer GCG and handling Editor declared their shared affiliation, and the handling Editor states that the process nevertheless met the standards of a fair and objective review.

## Footnotes

^1^www.schuhfried.at
